# Complete Genome Sequence of *Rhodococcus* sp. Strain 9, Isolated from Contaminated Australian Groundwater

**DOI:** 10.1128/mra.00317-22

**Published:** 2022-09-13

**Authors:** Varsha Naidu, Lachlan Hyde, Bart A. Eijkelkamp, Mallavarapu Megharaj, Suresh Subashchandrabose, Karl A. Hassan

**Affiliations:** a School of Environmental and Life Sciences, University of Newcastle, Callaghan, New South Wales, Australia; b ARC Centre of Excellence in Synthetic Biology, Macquarie University, North Ryde, New South Wales, Australia; c Molecular Sciences and Technology, College of Science and Engineering, Flinders University, Bedford Park, South Australia, Australia; d Global Centre for Environmental Remediation (GCER), University of Newcastle, Callaghan, New South Wales, Australia; University of Southern California

## Abstract

Here, we report the 7.7-Mbp genome sequence of *Rhodococcus* sp. strain 9, which was isolated from Australian groundwater contaminated with phenols and trichloroethylene. This strain has previously been shown to efficiently degrade *p*-nitrophenol and high-molecular-weight polycyclic aromatic hydrocarbons (PAHs).

## ANNOUNCEMENT

*Rhodococcus* is a Gram-positive genus classified in the phylum *Actinomycetota* (1). Species within this genus have an extraordinary catabolic versatility, allowing them to degrade several recalcitrant pollutants (1). Here, we present the genomic data of *Rhodococcus* sp. strain 9, which was isolated from Australian groundwater contaminated with phenols and trichloroethylene (2). Strain 9 has been shown to degrade and utilize *p*-nitrophenol (2) and low- and high-molecular-weight polycyclic aromatic hydrocarbons (PAHs), including phenanthrene, pyrene, and benzo[*a*]pyrene, as sources of carbon and energy (3, 4).

Strain 9 was grown in Luria-Bertani broth at 25°C with shaking (120 rpm) for 40 h. Total genomic DNA was extracted using the DNeasy UltraClean microbial kit (Qiagen), following the manufacturer’s instructions. The same extracted DNA was used for Illumina and Nanopore library preparation and sequencing at the Microbial Genome Sequencing Center (Pittsburgh, USA). Illumina sequencing libraries were prepared using the Illumina DNA prep kit and Integrated DNA Technologies 10-bp unique dual indexes; the libraries were sequenced on an Illumina NextSeq 2000 platform (2 × 151 bp), demultiplexed, and adaptor trimmed using bcl2fastq v2.20.0422. Oxford Nanopore sequencing libraries were prepared using Oxford Nanopore’s “Genomic DNA by Ligation” protocol and sequenced on a Nanopore R9 flow cell (R9.4.1) with no size selection, followed by signal processing, base calling, and adapter trimming using Guppy v4.2.2 (GPU mode). The Illumina reads were processed using fastp v0.22.0 (5) to remove adapter and barcode sequences, correct mismatched bases in overlaps, and filter low-quality reads. Nanopore long reads of <1 kb were removed using Filtlong v0.2.0 (https://github.com/rrwick/Filtlong). *De novo* hybrid assembly was performed with 166,391 filtered reads (*N*_50_, 11,298 bp) using Trycycler v0.3.3 (6). Briefly, 12 assemblies were generated using Flye v2.9-b1768 (7), Raven v1.2 (8), and Minipolish v0.1.2 (9), which predicted 3 contigs. The Trycycler v0.3.3 cluster function was used to group similar contigs from different assemblies into clusters (6). The Trycycler v0.3.3 reconcile function was used to reconcile each contig in each cluster, including rotating, trimming, and circularization, which resulted in two circular contigs and one linear contig (6). The Trycycler v0.3.3 functions msa, partition, and consensus were used to generate a consensus assembly, which was polished using Medaka v1.0.3, Bowtie2 v2.4.5 (10), and Pilon v1.24 (11). The linear contig was reassembled using Unicycler (Galaxy v0.4.8.0) (12) to recover the ends, which resulted in 691,102 bp. A BLASTn search of NCBI was performed (28 July 2022), using as the query 1 kbp of the right and left ends of the linear contig, which matched (query coverage, >95%; identity, >87%) the Rhodococcus jostii RHA1 linear plasmids pRHL2 (GenBank accession no. CP000433.1) and pRHL3 (CP000434.1). Collectively, this assembly predicted a circular 7,770,219-bp chromosome, a potentially linear 691,102-bp plasmid, and a circular 139,990-bp plasmid. Default parameters were used for all computational tools unless specified otherwise. Functional annotation of the genome was carried out using NCBI’s Prokaryotic Genome Annotation Pipeline (PGAP) v6.2 (13). Genome annotation predicted 8,095 genes in strain 9, of which 8,031 are coding DNA sequences and 64 genes encode RNA, including 12 rRNAs, 49 tRNAs, 2 noncoding RNAs (ncRNAs), and 1 transfer-messenger RNA (tmRNA).

The genome of strain 9 was compared with other *Rhodococcus* species. FastANI v1.3 (14) was used to compute the average nucleotide identity (ANI) against 99 *Rhodococcus* genomes in the RefSeq database (downloaded on 7 April 2022) which had “complete genome assembly” status. The organism with the highest ANI (98.0843%) to strain 9 was Rhodococcus opacus 1CP (BioProject accession no. PRJNA253567).

### Data availability.

The draft genome sequence is available at NCBI GenBank under the accession no. CP095403.1, and the raw sequencing reads are available at the NCBI Sequence Read Archive (SRA) under the BioProject accession no. PRJNA813342. The version described in this article is the first version.
